# Commentary: Lifetime Weight Characteristics of Adult Inpatients With Severe Anorexia Nervosa: Maximal Lifetime BMI Predicts Treatment Outcome

**DOI:** 10.3389/fpsyt.2021.775033

**Published:** 2021-11-08

**Authors:** Adrian Meule

**Affiliations:** ^1^Department of Psychiatry and Psychotherapy, University Hospital, Ludwig Maximilian University of Munich, Munich, Germany; ^2^Schoen Clinic Roseneck, Prien am Chiemsee, Germany

**Keywords:** anorexia nervosa, inpatient treatment, weight suppression, body mass index, OLS regression, repeated measures

In a recent study, Kaufmann et al. (1) examined predictors of weight change in adult inpatients with anorexia nervosa. Specifically, the dependent variable of interest—body mass index (BMI)—was available for three measurements: at admission, at discharge, and at follow up. Predictor variables of interest included lowest lifetime BMI, highest lifetime BMI, and weight suppression. Weight suppression refers to the difference between an individual's highest and current body weight and a higher weight suppression has previously been found to predict larger weight gain in both non-clinical and clinical samples (2–4). Data of 107 patients were analyzed. However, data of only 63 patients were available at follow up.

Besides other analyses reported in that article, the main analyses were linear, ordinary least squares (OLS) regression models. In such analyses, there can only be one dependent variable at once, so the authors chose to run three sets of analyses: several models for predicting BMI at admission, several models for predicting BMI at discharge, and several models for predicting BMI at follow up. The main results were—in line with previous findings—that higher weight suppression predicted larger weight gain. In addition, similar results were found with highest lifetime BMI, indicating that this variable is the key variable that drives effects of weight suppression. In other words, it is actually not necessary to compute the difference score of highest and current body weight but using highest body weight when controlling for current body weight suffices for predicting future weight gain, which is also in line with suggestions by others (5).

In this commentary, however, it is argued that there are several problems that result from this data analytic plan, which primarily stem from the fact that OLS regression does not suit such repeated measures data (e.g., with at least three measurements). First, at least three regression models need to be calculated to gain information that could be achieved in one single model when using more appropriate statistical techniques (thus, inflating Type I error rate). Second, weight trajectories across all three measurements cannot be modeled as the analyses only refer to one point in time (e.g., when predicting BMI at admission) or to changes from one point in time to the next one (e.g., when predicting BMI at discharge while controlling for BMI at admission). Third, when predicting BMI at follow up, the authors included both BMI at admission and BMI at discharge as predictor variables, which violates the assumption of independent observations for OLS regression. Fourth, because of missing data, the regression models for predicting BMI at follow up are based on a much smaller sample size than the other regression models, resulting in lower statistical power for detecting effects among other problems. Thus, results of the different models are not comparable.

A more appropriate statistical technique would be mixed-effects models, which are also often denoted as hierarchical linear models or multilevel models. Such models can solve all of the problems outlined above. To run such models, the data have to be in long format, that is, there would be three rows for each patient in the current example (one for each measurement) instead of one row for each patient and three columns for each measurement as in wide format. There are several free-of-charge software solutions with (e.g., JASP, jamovi) or without (e.g., the R-packages nlme or lme4) graphical user interfaces that can be used for running mixed-effects models. A crucial difference to OLS regression is that now the factor *time* (representing admission, discharge, and follow up in the current example) can be used as one of the predictor variables. Thus, a model that tests whether weight suppression predicts weight change across the three measurements would include BMI as dependent variable and at least three predictor variables: *time, weight suppression*, and the interaction term *time* × *weight suppression*. Thus, the effect of time would represent the general linear change in BMI across all three measurements and the effect of weight suppression would represent the association between weight suppression and BMI in general. If the interaction term is significant, this means that linear changes in BMI across measurements differ as a function of weight suppression. Importantly, missing data are included in the maximum likelihood estimation, so data of all 107 patients would be included in this model, although 44 patients did not provide BMI data at follow up.

Figure 2 in the article (1) suggests that weight changes are non-linear across measurements. That is, average body weight increased from admission to discharge but—on average—remained stable from discharge to follow up. In this case, a linear model may not fit the data appropriately. One possible solution for this would be to include a second-order polynomial of the time term, that is, *time*^2^. This quadratic time term then represents a non-linear change of body weight across measurements with one “bend” when visualizing the fit curve. Mirman (7) provides a hands-on guidance how to model such non-linear trajectories with the R-package lme4. A study that provides a practical example in which predictors of non-linear weight changes across admission, discharge, and follow up were examined with mixed-effects models in a sample of adolescent inpatients with anorexia nervosa has been published recently (6). In addition to solving OLS regression's problems when applied to repeated measures data, other techniques such as mixed-effects models or structural equation modeling also have further advantages related to the differentiation between fixed and random effects or between within- and between-person effects.

In conclusion, this commentary highlights that linear, OLS regression analyses should not be used when testing predictors of change over several measurements. While results found by Kaufmann et al. (1) are in line with previous studies (increasing confidence in their genuineness), interpretation of these findings is still limited by their data analytic strategy. Repeated measures data are complex and analyzing such data requires other techniques such a mixed-effects models or structural equation modeling. Using these techniques, changes over several measurements and their predictors can be modeled more appropriately as these have multiple advantages over OLS regression.

## Author Contributions

The author confirms being the sole contributor of this work and has approved it for publication.

## Conflict of Interest

The author declares that the research was conducted in the absence of any commercial or financial relationships that could be construed as a potential conflict of interest.

## Publisher's Note

All claims expressed in this article are solely those of the authors and do not necessarily represent those of their affiliated organizations, or those of the publisher, the editors and the reviewers. Any product that may be evaluated in this article, or claim that may be made by its manufacturer, is not guaranteed or endorsed by the publisher.

## References

[1] Kaufmann L-K Moergeli H Milos GF. Lifetime weight characteristics of adult inpatients with severe anorexia nervosa: maximal lifetime BMI predicts treatment outcome. Front Psychiatry. (2021) 12:682952. 10.3389/fpsyt.2021.682952 34335330 PMC8319499

[2] Gorrell S Reilly EE Schaumberg K Anderson LM Donahue JM. Weight suppression and its relation to eating disorder and weight outcomes: a narrative review. Eating Disord. (2019) 27:52–81. 10.1080/10640266.2018.1499297 30040543 PMC6377342

[3] Jenkins PE Lebow J Rienecke RD. Weight suppression as a predictor variable in the treatment of eating disorders: A systematic review. J Psychiatric Mental Health Nursing. (2018) 25:297–306. 10.1111/jpm.12462 29679513

[4] Lowe MR Piers AD Benson L. Weight suppression in eating disorders: a research and conceptual update. Curr Psychiatry Rep. (2018) 20:80. 10.1007/s11920-018-0955-2 30155651

[5] Schaumberg K Anderson LM Reilly EE Gorrell S Anderson DA Earleywine M. Considering alternative calculations of weight suppression. Eating Behav. (2016) 20:57–63. 10.1016/j.eatbeh.2015.11.003 26643591

[6] Meule A Schrambke D Furst Loredo A Schlegl S Naab S Voderholzer U. Inpatient treatment of anorexia nervosa in adolescents: a one-year follow up study. Eur Eating Disord Rev. (2021) 29:165–77. 10.1002/erv.2808 33230832

[7] Mirman D. (2014). Growth Curve Analysis and Visualization Using R. Boca Raton, FL: Chapman & Hall/CRC.

